# Monolithically Integrated Metasurface on a PCSEL for Depth Perception

**DOI:** 10.1021/acs.nanolett.5c02540

**Published:** 2025-07-11

**Authors:** Wen-Cheng Hsu, Wen-Chien Miao, Yu-Heng Hong, Hao-Chung Kuo, Yao-Wei Huang

**Affiliations:** † Department of Photonics, College of Electrical and Computer Engineering, 34914 National Yang Ming Chiao Tung University, Hsinchu 300093, Taiwan; ‡ Semiconductor Research Center, Hon Hai Research Institute, Taipei 11492, Taiwan

**Keywords:** metasurfaces, photonic crystal surface emitting laser, monolithic integration, on-chip dot projector, in situ fabrication, structured light, depth sensing

## Abstract

Dot projectors are pivotal for depth perception in modern consumer electronics, from smartphones to extended reality devices, enabling applications in computational imaging, machine vision, and privacy-preserving technologies. However, existing dot projector designs face significant challenges related to their size and power consumption. Here, we demonstrate the first monolithic integration of a metasurface hologram and a photonic crystal surface-emitting laser (PCSEL) to realize a chip-scale structured light projector. This approach achieves unprecedented reductions in both device footprint and power usage, while preserving practical 3D sensing capabilities. Our wafer-level design features a compact footprint of 0.025 mm^3^, representing an approximately 2450-fold reduction in volume compared to commercial DOE-VCSEL dot projectors, while also reducing power consumption by 28.7%. The integration strategy offers promising fabrication compatibility and represents a transformative advancement in a compact transceiver system, paving the way for next-generation applications in biometrics, extended reality, and consumer electronics.

## Graphical abstract

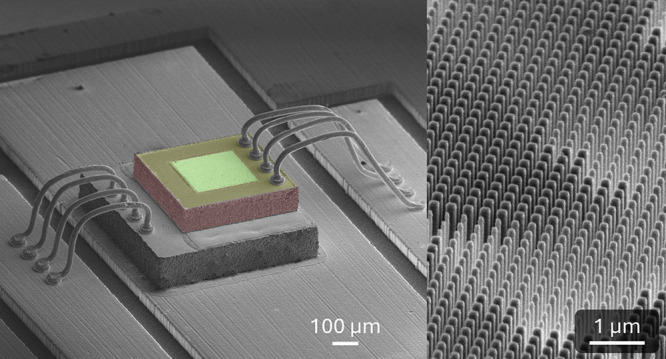

Structured light has become a foundational technique for depth sensing in consumer electronics, notably in Apple’s Face ID system.^1–4^ By projecting patterned light onto a surface and analyzing its distortion with a near-infrared camera, this method enables accurate three-dimensional (3D) reconstruction. It supports critical applications such as facial recognition and extended reality (XR), where high-resolution, reliable depth perception is essential. Apple’s Face ID system relies on a dot projector that generates over 32,000 structured light dots using a 4F optical imaging system. This 61.25 mm^3^ module integrates a vertical-cavity surface-emitting laser (VCSEL) array (∼366 emitters), dual lenses, a folding waveguide, and a diffractive optical element (DOE).^2,4,5^ The lens system magnifies the VCSEL emission, and the DOE diffracts it into a dense dot pattern. Despite its effectiveness, the 4F architecture imposes strict spatial constraints, limiting further miniaturization (see Figure S1). Furthermore, the high power consumption of the VCSEL array poses challenges for wearable and energy-efficient applications.

Metasurfaces, subwavelength nanostructures capable of manipulating phase, amplitude, and polarization, offer a promising path toward ultracompact optics.^6,7^ By replacement of bulky components, they enable unparalleled miniaturization and advanced functionality. Recent studies have explored metasurface-enabled structured light for depth sensing, achieving higher dot counts and wider field of view (FOV). However, these designs often depend on bulky solid-state lasers,^8–15^ or integrate only single-emitter VCSELs,^16–21^ resulting in systems that are either too large or underpowered for practical deployment.

Notably, Metalenz’s 18K Meta-optic integrates a metasurface with a 391-emitter VCSEL array within a 775 μm wafer thickness, reducing device volume to 21.16 mm.^22–25^ Yet, its hybrid 4F system still limits thickness scaling due to focal length constraints, preventing full monolithic integration at the chip scale. These constraints underscore the need for innovative approaches that combine metasurfaces with advanced light sources to achieve compact, efficient, and fully integrated depth sensing systems.

To address these challenges, in this study, we present a monolithically integrated metasurface-PCSEL chip that achieves robust depth sensing with unprecedented miniaturization and power efficiency. As shown in Figure 1(a), our design departs from the bulky 4F architecture by integrating a metasurface directly with a single PCSEL, whose narrow-divergence beam enables efficient dot projection without additional optics. PCSELs, utilizing band-edge resonances, provide higher coherence, large-area emission, and superior beam quality compared to other semiconductor lasers, making them well-suited for metasurface integration.^26–30^ This monolithic integration eliminates the reliance on external light sources, drastically shrinking the system footprint and reducing the energy efficiency. Figure 1(b) compares the size of our chip with the iPhone dot projector, demonstrating its ultracompact 0.025 mm^3^ volume. Figure 1(c–e) illustrates the evolution from traditional 4F systems to hybrid metasurface system (e.g., Metalenz) and finally to our fully integrated chip-scale solution. The compactness and simplicity of our platform make it ideal for integration into next-generation wearable devices, such as XR glasses.^31,32^

**Figure 1:**
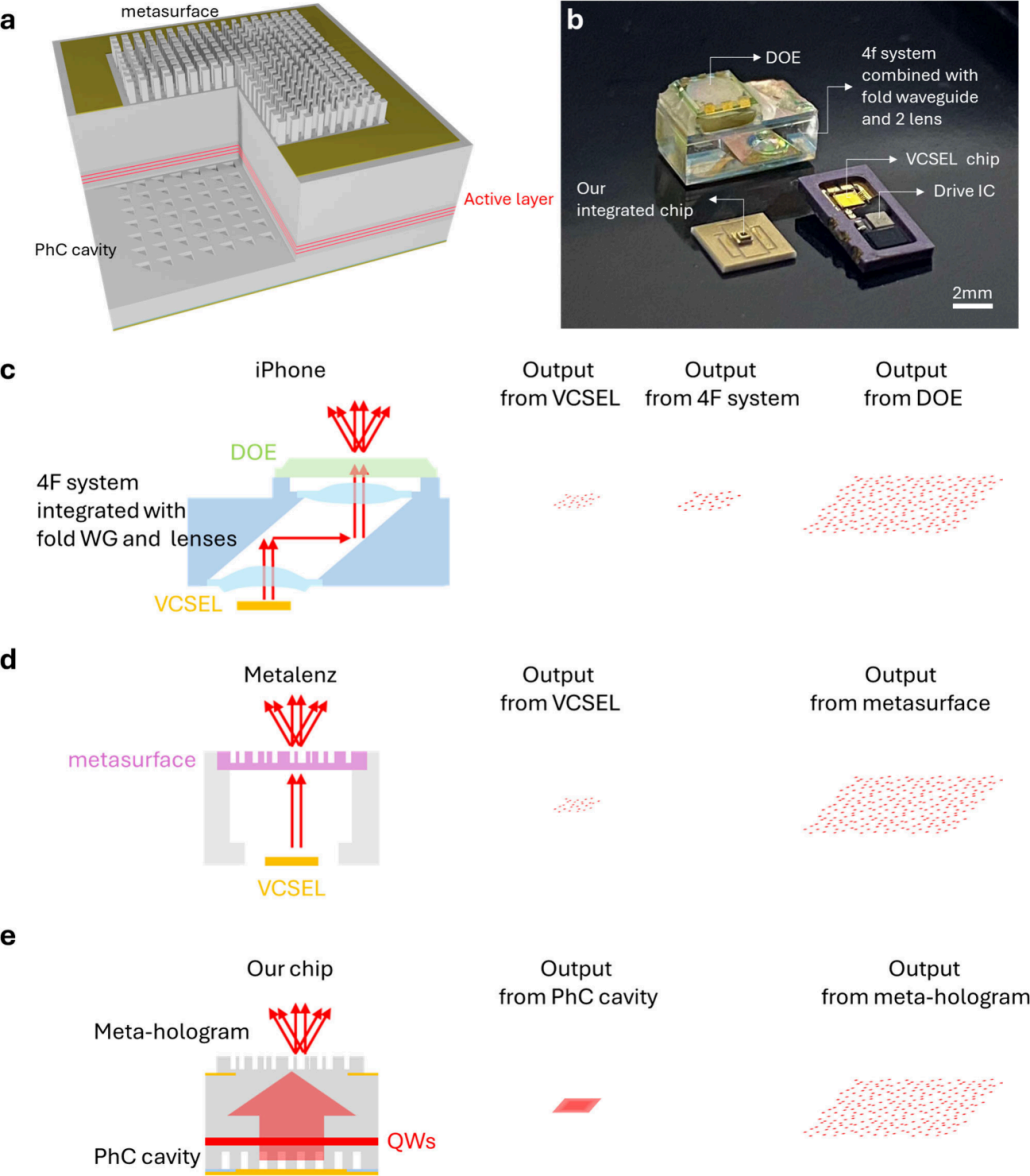
Structured light projector designs. (a) A 3D illustration of the monolithically integrated on-chip structured light projector with the metasurface fabricated in situ on the PCSEL. (b) A size comparison between our integrated chip, mounted on a carrier and packaged as an SMD device, and the discrete components of the commercial dot projector (Face ID of iPhone X, Apple). (c) Schematic architecture of a commercial iPhone dot projector. (d) Schematic architecture of Orion dot projector (ML1DP18MS, Metalenz). (e) Our proposed dot projector architecture.

Photonic crystal (PhC) modes operating around the second-order gamma (Γ_2_) point of the photonic band edge serve as laser cavity modes in PCSELs, exhibiting strong in-plane resonance and efficient out-of-plane emission.^26^ These modes offer excellent beam directivity, low divergence, and high output power, especially when designed with asymmetric PhC air holes.^27,28^ While prior studies have demonstrated metasurface holograms using PCSELs,^29,30^ they primarily assumed long propagation distances. The impact of short propagation—such as that constrained by the substrate thickness—remains underexplored.^33^ To address this, we implemented a hybrid simulation framework to evaluate metahologram-integrated PCSELs under compact configurations, enabling a more realistic analysis of their optical performance and integration feasibility.

First, the Γ_2_ point of the PhC mode is generated by calculating the band structure of a periodic triangular air hole in GaAs. Parameters derived from the calculated band structure were used to construct a finite 2D PhC structure with 100 periods along each in-plane direction, yielding the corresponding band edge mode. The PhC setup, band structure, and electric field distribution of the PhC mode are illustrated in Figure 2(c–e). In our simulation, the lattice constant of the PhC is set to 287 nm. Triangular air holes (inset of Figure 2(c)) are employed to achieve a dot-shaped beam profile, enhanced output power, and an expanded process tolerance window. The band structure near the Γ_2_ point, highlighted by the red dashed range in Figure 2(c), is magnified in Figure 2(d), revealing the A, B, C, and D modes. Field simulations consistently indicate lasing in the B mode due to its lowest cavity loss. The corresponding field distribution of the B mode is presented in Figure 2(e).

**Figure 2:**
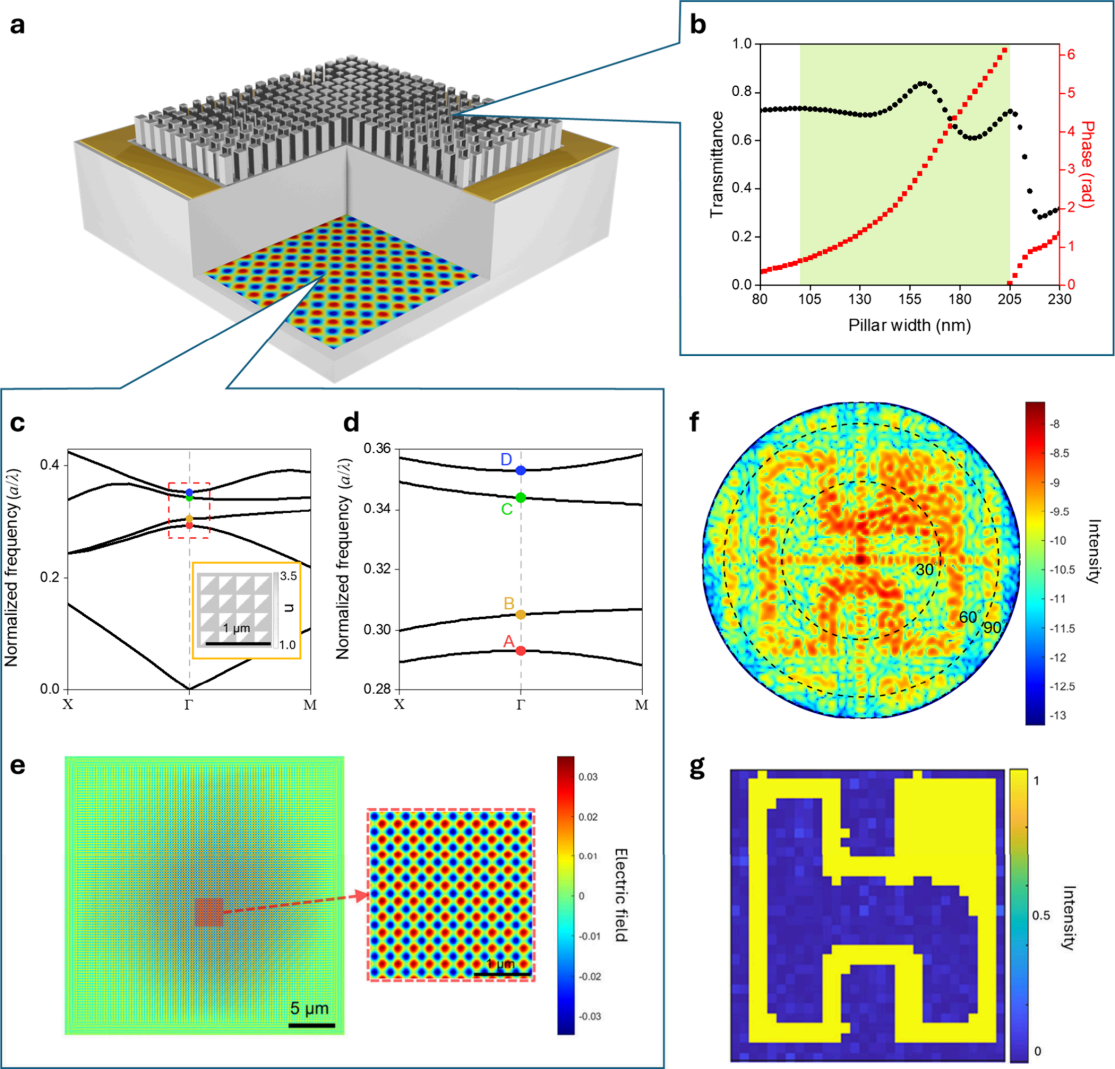
Illustration of the hybrid simulation for the monolithic integration of a structured light projector and corresponding simulation results. (a) 3D schematic of the hybrid simulation setup for monolithic integration, featuring a GaAs metasurface hologram placed on a substrate with a PhC mode as the light source beneath the metasurface. (b) Schematic of the meta-hologram design generated using the GS algorithm and a meta-atom library with a propagation phase design strategy. (c) Band structure of the GaAs PhC with triangular air holes, calculated using a finite difference eigenmode (FDE) solver in Lumerical. The inset shows the 2D setup of the PhC structure. (d) Magnified view of the band structure around the Γ_2_ point from (c). (e) Electric field distribution of a finite-size PhC mode simulated using Lumerical FDTD. (f) Far-field projection of the Hon Hai logo obtained via the hybrid simulation method. (g) Numerical projection of the Hon Hai logo calculated by using the GS algorithm.

The metasurface hologram, shown atop the PCSEL in Figure 2(a), is composed of square pillars (meta-atoms) of varying dimensions. It was designed using a meta-atom library (Figure 2(b)) in conjunction with the holographic phase distribution derived from Gerchberg–Saxton (GS) algorithm.^34,35^ We select 43 meta-atoms with widths ranging from 100 to 205 nm, covering the full 2π phase range. This design range, highlighted in green in Figure 2(b), represents a trade-off between phase coverage, transmission efficiency, and fabrication constraints. Each pixel of the hologram is composed of a 2 × 2 array of meta-atoms, each with a 260 nm period. Separated by a gap of about 10 μm, the metasurface hologram manipulates the near-field wavefront from the PhC mode, transforming it into arbitrary far-field patterns. Furthermore, the 2 × 2 meta-atom array leads to a larger zero-order holographic image, thereby improving the brightness. More details of hybrid simulation setup are seen in Figure S2. Here, we used the “Hon Hai” logo to verify our simulation workflow. The far field of FDTD results, shown in Figure 2(f), is compared with the GS algorithm results in Figure 2(g). It is evident that the Hon Hai logo is clearly resolved in the FDTD results and closely matches the numerical calculations.

To numerically characterize the far-field pattern, we evaluated finite 2D PhC modes of varying footprints (by changing the number of periods; see Figure S3). We then designed a metasurface to generate a 5 × 5 dot array and observed how the diffraction spot size varies accordingly. Each dot in the PhC mode shows a beam divergence of roughly 0.5–6°, corresponding to 100–30 periods in each in-plane direction. The metasurface’s phase map covers an area of 19.8 × 19.8 μm^2^, yielding a pixel sampling angle (i.e., angular resolution in k-space) of about 3.03°. Simulation results indicate that the far-field divergence angle of the diffracted dots is determined primarily by the size of the PhC mode rather than by the metasurface dimensions.

The monolithic metasurface-PCSEL integrated device was fabricated using a strategically designed flip-chip process aimed at reducing fabrication complexity and overall cost. A similar approach has also been demonstrated in monolithic metasurface–VCSEL integrated devices.^16–21^ The general epi-wafer fabrication of PCSELs involves a regrowth process to embed the PhC structure, which is associated with a low yield and high costs. However, regrowth-free PCSEL process is also published for more efficiency mass production.^36–40^ Here, we employed a regrowth-free PCSEL architecture with a flip-chip process to integrate PCSEL and the metasurface. The PhC structure is formed by lithography and directly deep etching in the p-contact layer and cladding layer. After flipping and polishing the substrate via chemical-mechanical polishing (CMP), the metasurface was fabricated on the n-side of the substrate. The distance between the metasurface and PhC cavity is determined by the polished substrate thickness (about 100 μm). Further details of the fabrication process are provided in the Methods and in Figure S4.

In Figure 3(a–b), we present the light–current–voltage (L–I–V) characteristics and emission spectrum of the integrated device, measured under a 5% duty cycle and a 1 μs pulse width. The solid curves correspond to the metasurface-integrated PCSEL, while the dashed curves represent the bare PCSEL. As shown in Figure 3(b), the metasurface-integrated PCSEL operates in single-mode at a wavelength of 938.5 nm when driven at 0.3 A. The slope efficiency of the light–current (L–I) curve—defined as the optical power output per unit of injected current above the lasing threshold—is a key performance metric for semiconductor lasers. Here, the measured slope efficiencies with and without the metasurface are 140.6 and 142.2 mW/A, respectively. This close match arises because the meta-atoms’ transmittance is nearly the same as that of a GaAs–air interface, which is 66–85% by simulation (see Figure 2(b)) and 69% in theory.^41^ Experimentally, the metasurface exhibits an absolute transmittance of 68.3%—obtained from the ratio of the slope efficiencies—closely matching the theoretical GaAs–air interface transmittance. Moreover, the threshold current of the device remains comparable between the two configurations, indicating that the integration of the meta-atoms on the substrate side does not adversely impact the PCSEL cavity performance. Since cavity feedback or optical coupling effects require subwavelength spacing and high reflectivity of metasurfaces,^42–44^ our approach—with a gap of approximately 100 wavelengths between the metasurface and the photonic crystal—effectively avoids such issues. The inset images of Figure 3(b) are the optical microscope (OM) images of our devices. The square green area on top of PCSEL indicates the location of the metasurface hologram. The scanning electron microscope (SEM) images of our sample are shown in Figure 3(c) and the enlarged metasurface region is in Figure 3(d); our device has a volume of 500 × 500 × 100 μm^3^ and a metasurface area of 300 × 300 μm^2^. The meta-atoms are designed as a square pillar with 700 nm height with center-to-center distance of 260 nm. This wafer-level optics leads to our chip volume being 2450 times smaller than the commercial dot projector in Face ID system (iPhone 15, Apple) and 846 times smaller than the metasurface-VCSEL integrated dot projector (Orion, Metalenz).

**Figure 3:**
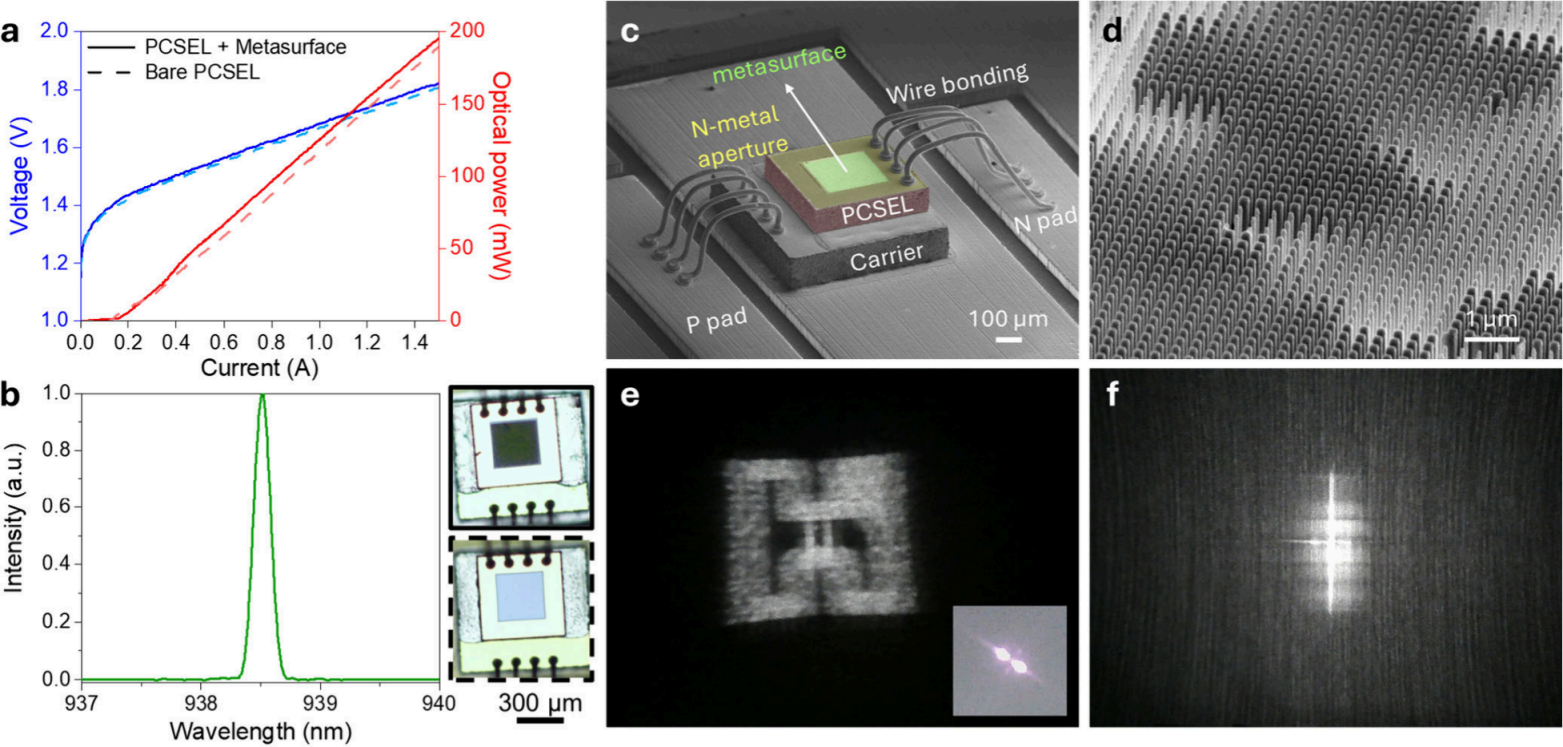
Device characteristics, SEM images, and optical performance. (a) The output optical power, current, and voltage (L–I–V) curves of the PCSEL with metasurface (solid) and bare PCSEL (dashed). (b) Emission spectrum of the metasurface-integrated PCSEL. The inset shows OM images of the integrated device (top) and bare PCSEL (bottom). (c) SEM image of the integrated device highlighting the metasurface region. (d) Magnified SEM view of the metasurface hologram within the central green region of (c). (e) Reconstruction of the “Hon Hai” logo projected by the integrated device. The inset shows the beam shape of the PCSEL without the metasurface. (f) Reconstruction of random-dot pattern after propagation over 70 cm.

During the initial phase of conceptual verification, the metasurface was designed to project a holographic image of the Hon Hai logo using a propagation phase strategy. The Hon Hai logo generated by the structured light projector was successfully projected onto paper and captured using a webcam. The device operated with a 5% duty cycle, a pulse width of 1 μs, and a driving current of 300 mA. Obviously, the beam profile of bare PCSEL was effectively transformed to replicate the Hon Hai logo, as illustrated in Figure 3(e), highlighting the capability of the metasurface-PCSEL integration to produce intricate holographic patterns. However, the reconstructed image of the Hon Hai logo appeared to be blurred. This issue arises because the experimental PhC mode from PCSEL does not excite in the correct Γ_2_ point,^38,45^ which may primarily result from the direct contact between the metal and the PhC layer. As a result, an x-shaped (or cross-shaped) pattern with two distinct spots appears in the far field, as shown in the inset of Figure 3(e). This effect effectively splits the projection of the Hon Hai logo into two tiny angular displacements, causing the two overlapping images to produce a blurred reconstruction with noticeable artifacts in the zero-order spots.

Random dot patterns were encoded into the metasurface hologram with a minimum pixel spacing of 6 between each dot in the ideal holographic image, corresponding to the dot sampling angle of 1.542 degrees.^30^ This design effectively accommodates the divergence angle of the laser (∼1°, inset of Figure S5­(a)) while preserving the clarity and fidelity of the projected dot patterns, thereby ensuring robust performance for structured light applications. To assess the depth sensing capability of bare metasurface hologram, its reconstructed structured light pattern was initially tested with a commercial PCSEL prior to their integration into the on-chip device. Detailed results from these depth sensing evaluations are provided in Figure S5. Additionally, we investigate the depth sensing with on-chip approach. The experimentally reconstructed structured light pattern, shown in Figure 3(f), was obtained under the same conditions as those in the previous tests, except for an operating current of 1.2 A. The projected image was captured on a flat screen positioned 70 cm away, simulating the intended application in depth sensing. The reconstructed dot images exhibited a convolution with a cross-shaped pattern and transitioned from random dots to quasi-random lines, attributed to imperfections in the PhC mode as well as the holographic image shown in Figure 3(e). Although the reconstructed dot images were blurred and not sharply defined, the system still demonstrated functionality in depth perception, as discussed in the next section.

Our depth sensing system comprises a standard webcam, an integrated dot projector chip, and connection with a laser controller; the full system is shown in Figure S6. The integrated device was mounted next to the webcam using a custom gray fixture, ensuring a baseline *b* separation of approximately 1.5 cm between the two components. The schematic setup is depicted in Figure 4(a), while Figure 4(b) presents a photograph of the actual system. The integrated device was operated under conditions replicating the far-field characteristics of the dot pattern with structured light projected onto a screen positioned 70 cm away. This distance serves as the reference distance *z*_ref_ for the depth perception algorithm, and the dot image serves as the reference image.

**Figure 4:**
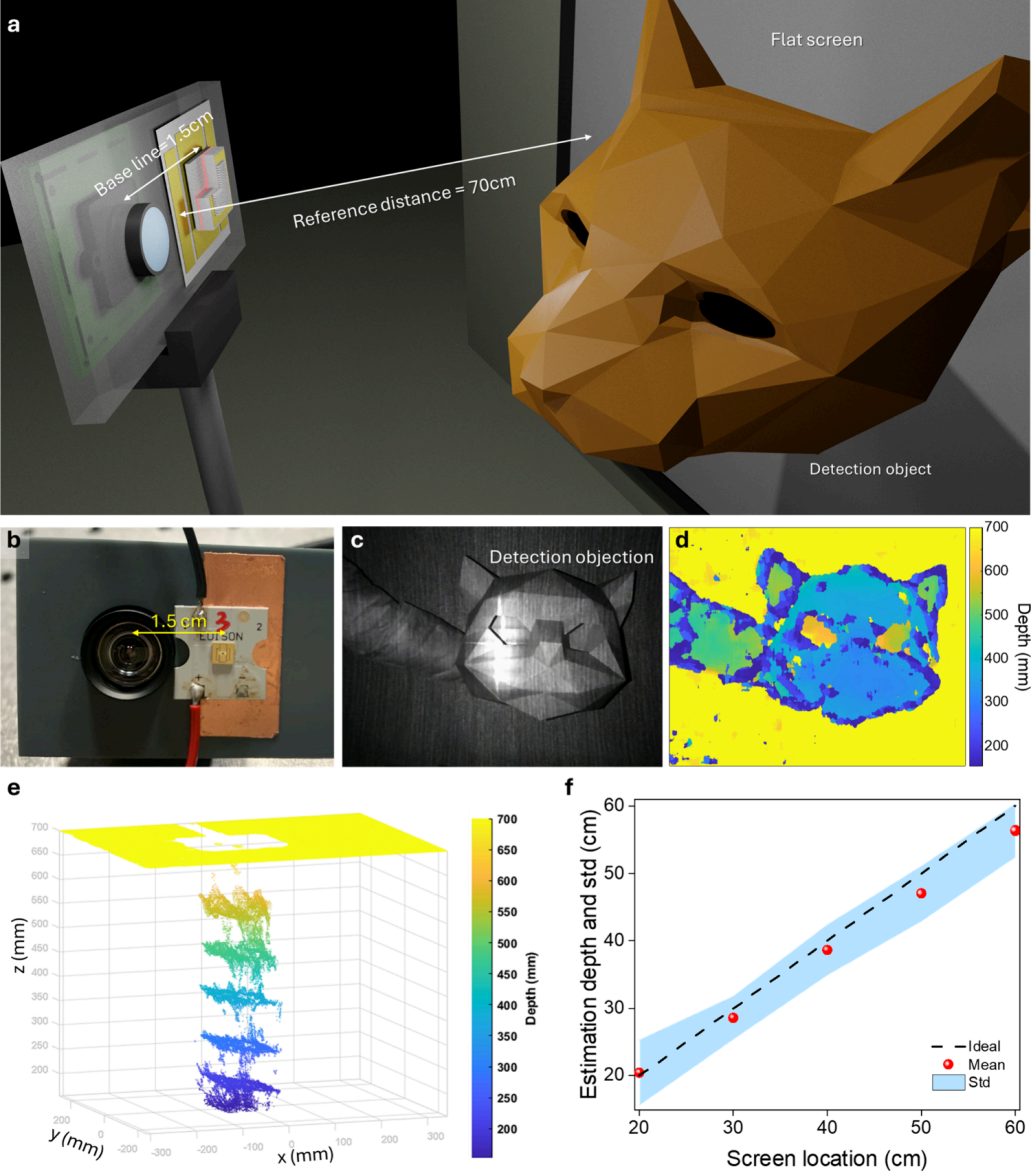
Single-shot depth sensing demonstration by combining our integrated chip and webcam. (a) Single-shot depth sensing setup. (b) Captured image with a hollow cat helmet. (c) Depth image corresponding to the captured image in (b). (d) Corresponding 3D point cloud of (c). (e) Overlay of 3D point cloud of flat board in different position. (f) Curve of the ideal position and real measured position.

The target object for our depth perception system is a cat-shaped helmet with dimensions of 28 × 30 × 29.2 cm^3^, held by hand, as shown in Figure 4(c). A second dot image was captured with structured light projected onto the object. By estimation of the disparity between the reference dot image and the captured image, a disparity map was generated. This map was then transformed into depth information using a triangulation-based approach, as described in prior studies.^30,46,47^ The depth is calculated using the equation:

1 $$D\left(x,y\right)=z_{ref}{\left\{1+z_{ref}\left[\frac{d\left(x,y\right)}{f_{x}\times b}\right]\right\}}^{-1}$$

where *D*(*x*,*y*) is estimated depth, *d*(*x*,*y*) is estimated disparity (in pixels), and *f*_*x*_ is the focal length of webcam (in pixels) determined through precalibration. The corresponding depth map for the cat helmet is presented in Figure 4(d). It clearly reveals the hand positioned behind the helmet as well as hollow regions in the eyes, which are approximately 30 cm deeper than the helmet’s surface. In addition, the power consumption of our device is 104.3 mW, which is significantly lower than that of commercial VCSEL-array-based dot projectors, as shown in Table 1.

**Table 1:** Comparison of Our Device and State-of-the-Art with Impact Parameters

	Face ID of iPhone X, Apple^2,3,5^	Face ID of iPhone 15, Apple^2,4,5^	Orion, Metalenz^22^	ref 16	ref 17	ref 30	our device
laser type	VCSEL array	VCSEL array	VCSEL array	single emitter VCSEL	single emitter VCSEL	single PCSEL	single PCSEL
diffractive optics	DOE	DOE	metasurface	metasurface	metasurface	metasurface	metasurface
dimension of device (mm)	5.0 × 3.5 × 6.0	5.0 × 3.5 × 3.5	2.7 × 2.5 × 3.135	0.1 × 0.1 × 0.625 × π	0.043 × 0.043 × 0.625 × π	-	0.5 × 0.5 × 0.1
volume of device (mm^3^)	105	61.25	21.161	1.963 × 10^–2^	3.631 × 10^–3^	-	2.50 × 10^–2^
operating peak current (A)	2.5	2.5	2.0	2.0 × 10^–4^	5 × 10^–3^	0.3	1.200
operation voltage (V)	1.95	1.95	2.1	1.50	-	1.6	1.738
duty cycle (%)	3	3	10	100	100	100	5
pulse width (ms)	1	1	0.1	CW	CW	CW	0.001
power consumption (mW)	146.25	146.25	420	0.30	-	480	104.28
peak optical output power (W)	2.15	2.15	1.5	1.00 × 10^–5^	-	0.0385	3.0847
structured light	yes	yes	yes	no	yes	yes	yes
depth sensing ability	yes	yes	yes	no	no	yes	yes

To further evaluate the depth sensing capabilities of the system, a standard flat board (EDU-VS1, Thorlabs) was measured at various distances ranging from 20 to 60 cm. The depth data were visualized as 3D point clouds, demonstrating consistent reconstruction at 10 cm intervals, as shown in Figure 4(e). However, the central region of the point cloud exhibits missing information, which can be attributed to the excessive intensity of the zero-order spot and the low texture features in that area, negatively impacting the reconstruction accuracy. A similar issue was observed in the depth measurement of the cat helmet, although it is not readily visible in the 2D depth map (*x*-*y* view) presented in Figure 4(d). Figure 4(f) provides an analysis of the measurement results from Figure 4(e), including the measured depth (red points) versus the ground truth (black dashed line) and the standard deviation (blue-shaded region). The measured depth aligns closely with the ideal curve with an average standard deviation of approximately 7.84 cm. These experiments demonstrate that despite the structured light pattern being subject to distortion and blurring, the system maintains sufficient depth sensing capabilities.

We further evaluated the depth sensing performance using a normal random dot structured light pattern, integrating an individual metasurface hologram (identical in design to the on-chip case) with a Gaussian-like light source from a commercially available PCSEL (L13395-04, Hamamatsu). The reconstructed dot image exhibited improved performance, as illustrated in Figures S7­(a) and S7­(b). This improvement resulted in enhanced depth detection quality, particularly along the object boundaries, enabling continuous depth detection of the cat helmet (Figure S7­(c)). When the depth sensing performance was assessed on a flat board, the average standard deviation was approximately 5.33 cm (Figure S8), reflecting only a modest improvement over the on-chip case.

This study presents a groundbreaking monolithic integration of a metasurface and a PCSEL, achieving unprecedented compactness and efficiency in structured light projectors for depth sensing applications. Despite the challenges posed by blurred dot patterns due to improper PhC mode excitation, the system successfully demonstrated depth sensing capabilities. The Supporting Information includes a detailed comparison and analysis of meta-holograms integrated with PCSELs under both normal Γ_2_ modes and abnormal conditions, showcasing clear dot projection functionality in standard scenarios. Furthermore, polarization-sensitive metasurfaces can also be monolithically integrated on our PCSEL-based platform, provided that proper polarization alignment is ensured during fabrication. Our previous studies on metasurfaces designed with geometric phase strategies further supports PCSEL’s suitability as a robust, efficient, and compact platform for metasurface integration.^48^

We compare our approach to state-of-the-art technologies, with specifications detailed in Table 1. Compared to DOE-VCSEL and metasurface-VCSEL integrations, our monolithically integrated metasurface-PCSEL system delivers significant advancements in size. The device boasts an ultracompact footprint of 0.025 mm^3^, representing a 2450-fold and 846-fold volume reduction compared to commercial 4F-based dot projectors. Enabled by flip-chip integration, our system advances beyond previous metasurface-PCSEL assemblies by scaling from module-level packaging to chip-level integration.^30^ While the volume is slightly larger than that of refs 16 and 17, those designs lack depth sensing capabilities.

The chip-scale solution eliminates system complexity and enhances the practical applicability. PCSELs, with their superior optical properties, such as high-power output and low divergence, address the limitations of VCSEL-based systems, including low output power and high divergence. Additionally, the integration of PCSELs significantly reduces power consumption by over 28.7% and 75.2% compared to those from the DOE-VCSEL array packaged device and the metasurface-VCSEL array packaged device. As demonstrated in the device’s *I*–*V* characteristics, our approach enables seamless adaptation to existing commercial architectures.^49,50^

The compact and energy-efficient design is especially critical for wearable devices, facilitating seamless integration into XR glasses and extending the battery life. This study establishes a new benchmark for miniaturized photonic systems, paving the way for the next generation of consumer electronics and setting the stage for broader applications in both structured-light and flash LiDAR depth-sensing technologies.

## Materials and Methods

### Fabrication Process

Our p-side down integration process was developed with Phosertek Tech., which provided the epi-wafer and PhC cavity fabrication process in the p-side, and we followed fabrication of the meta-hologram in the n-side substrate. The fabrication process begins with electron beam lithography (e-beam lithography) to define a 300 × 300 μm^2^ PhC cavity pattern, followed by deep etching using reactive ion etching with inductively coupled plasma (RIE-ICP). After the PhC structure is formed, isolation trenches are patterned and etched. Subsequently, a 250-μm-diameter oxide aperture is created above the PhC structure. The p-contact metal is then deposited across the entire p-side surface via sputtering, and the substrate thickness is reduced to 100 μm by CMP.

After the p-side processes are completed, a double-sided alignment of deep ultraviolet (DUV) lithography is conducted. Alignment keys in 800 nm height are patterned and etched on the n-side via double-sided alignment and RIE-ICP to facilitate precise alignment for subsequent e-beam lithography. Using a three-point alignment method with the n-side alignment keys, a 270 × 270 μm^2^ metasurface pattern is defined within the PhC cavity region. The metasurface with a height of 700 nm is then formed through RIE-ICP etching. To avoid mismatch alignment between laser polarization and metasurface directivity causing efficiency drop, we design metasurface in propagation phase strategy.

The last semiconductor process is metal aperture lift-off, which uses DUV patterning, deposition of n-contact metal via sputtering, and PG remover to open a contact window in the metasurface region. Then, the wafer is diced into 500 × 500 μm^2^ chip size after annealing. The fabricated chips are packaged using surface-mount device (SMD) packaging for wide field-of-view (FOV) emission. The details of the whole fabrication process and process flow are shown in the Supporting Information.

## Supplementary Material



## Data Availability

All data generated or analyzed during this study are included in this published article.

## Notes

Additional details on the composition of commercial dot projectors, the hybrid simulation framework, far-field results from hybrid diffractive simulations, the fabrication flow of the monolithically integrated device, structured-light projections generated using various PhC beam profiles, divergence angles of different PCSEL samples, the real-world depth sensing setup, and a comparative analysis of reconstructed patterns and depth sensing performance between normal and abnormal structured-light projections (PDF)
