# Supplementary material for: Monolithically Integrated Metasurface on a PCSEL for Depth Perception
Source: Nano Lett. 2025 Jul 11;25(29):11382–90. doi: 10.1021/acs.nanolett.5c02540 (PMC12291584; doi:10.1021/acs.nanolett.5c02540)
Supplement: Supplementary file 1 [file nl5c02540_si_001.pdf]

Supporting Information for

Monolithically Integrated Metasurface on a PCSEL for Depth Perception

Wen-Cheng Hsu,[1,2] Wen-Chien Miao,[1,2] Yu-Heng Hong,[2,*] Hao-Chung Kuo,[1,2,*] and Yao-Wei Huang[1,*]

1. Department of Photonics, College of Electrical and Computer Engineering, National Yang Ming Chiao Tung University, Hsinchu 300093, Taiwan

2. Semiconductor Research Center, Hon Hai Research Institute, Taipei 11492, Taiwan

*Address correspondence to: enoch.yh.hong@foxconn.com (Y.-H.H.); hckuo0206@nycu.edu.tw (H.-C.K.); and ywh@nycu.edu.tw (Y.-W.H.)

Contents

Figure S1. Composition of commercial dot projectors.

Figure S2. Hybrid simulation framework and setup for the PCSEL and metasurface.

Figure S3. Far-field results from hybrid diffractive simulations under varying PCSEL divergence angles.

Figure S4. Fabrication process flow of the monolithically integrated metasurface-PCSEL device.

Figure S5. Structured-light projections generated using various PhC beam profiles illuminating the same metasurface hologram.

Table S1. Divergence angles of different PCSEL samples.

Figure S6. The real-world depth-sensing setup.

Figure S7. Comparison of structured-light projections between normal and abnormal cases.

Figure S8. Comparison of depth-sensing performance between normal and abnormal structured-light projections.

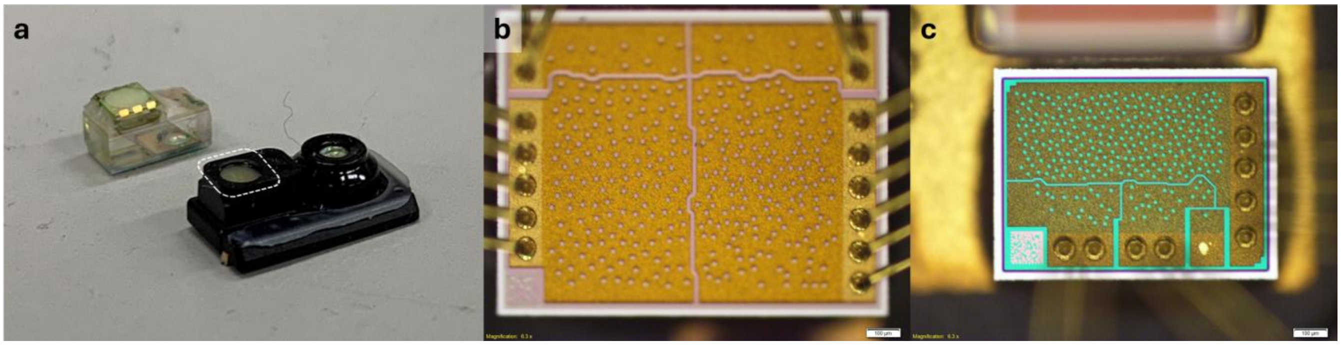

Figure S1. Composition of commercial dot projectors. a. Optical modules of dot projectors: the transparent module is from the iPhone X, and the white dashed region in the black module highlights the dot projector section of the iPhone 15. **b.** VCSEL array used in the iPhone X dot projector, exhibiting a random emitter distribution. **c.** VCSEL array used in the iPhone 15 dot projector, illustrating a more structured emitter layout.

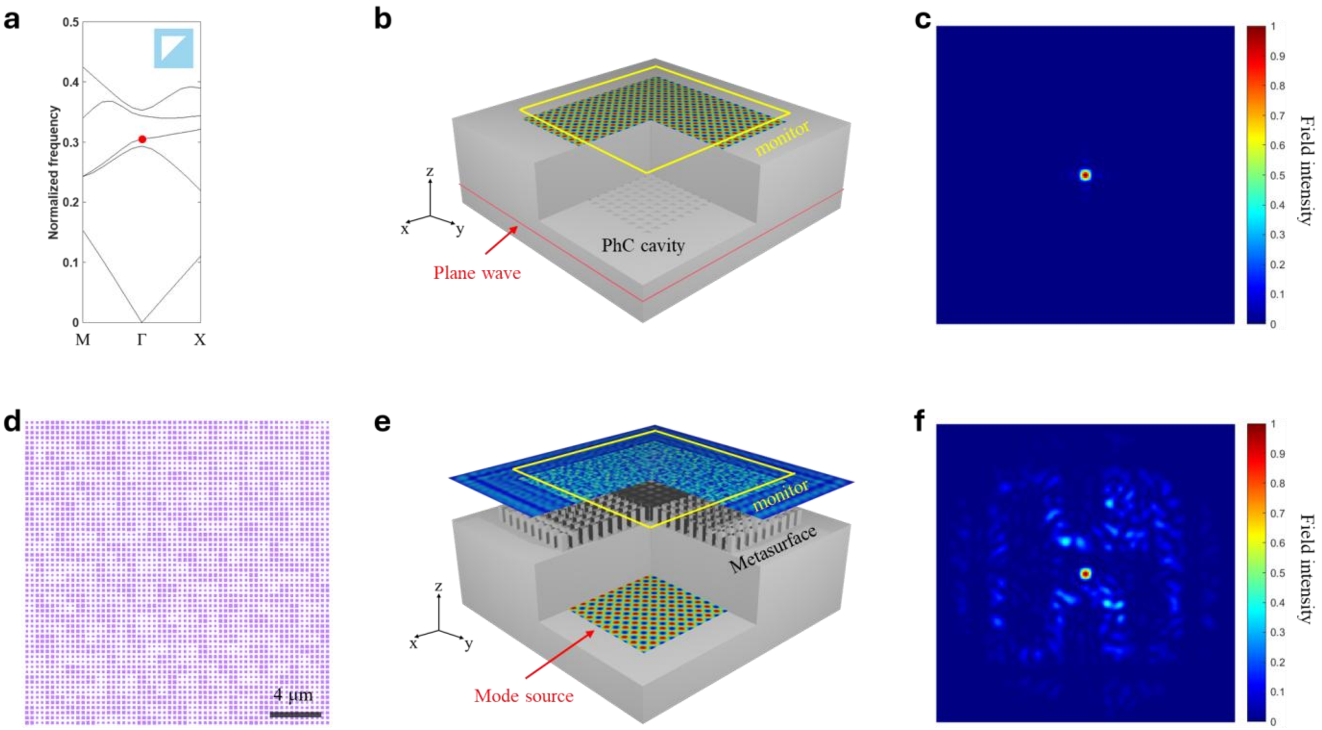

Figure S2. Hybrid simulation framework and setup for the PCSEL and metasurface. a. Photonic band structure of the PCSEL near the Γ_2 point, used to determine the lattice constant for targeting the B mode wavelength (marked by the red dot). **b.** Finite-size PCSEL simulation setup for generating the PhC mode. The red region indicates a plane wave source used to excite the PhC mode through the cavity. The yellow region marks the field monitor. **c.** Far-field pattern of the PhC mode corresponding to the setup in (b). **d.** Metasurface hologram layout designed to display the Hon Hai logo, created using a meta-atom library and the GS algorithm. The varying nanopillar widths encode the phase distribution. **e.** Hybrid simulation combining the PhC mode with the metasurface structure. The near field recorded by the monitor is used for far-field transformation. **f.** Far-field result from the hybrid simulation, showing the evolution from the single-spot PhC beam (c) into the Hon Hai logo pattern.

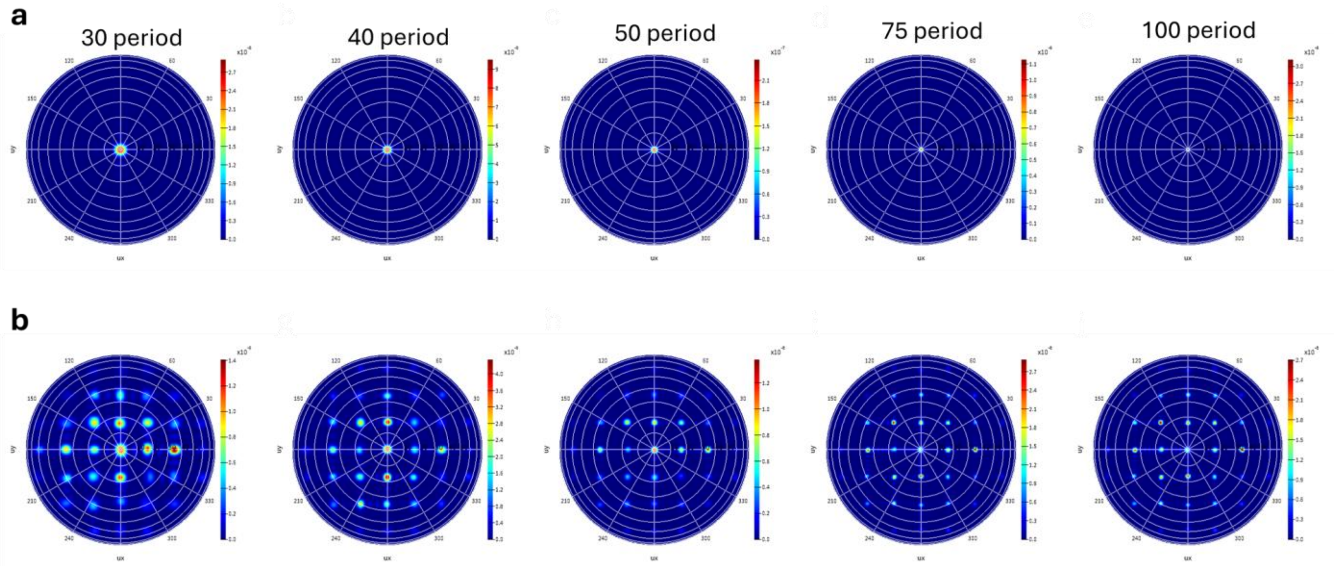

Figure S3. Far-field results from hybrid diffractive simulations under varying PCSEL divergence angles. a. Simulated PCSEL far-field profiles with divergence angles of 6°, 4°, 2°, 1°, and 0.5°, respectively. **b.** Simulated metasurface diffraction patterns forming a 5 × 5 dot array, corresponding to the divergence angles shown in (a). The size and clarity of the diffracted spots vary as a function of the input PhC beam divergence.

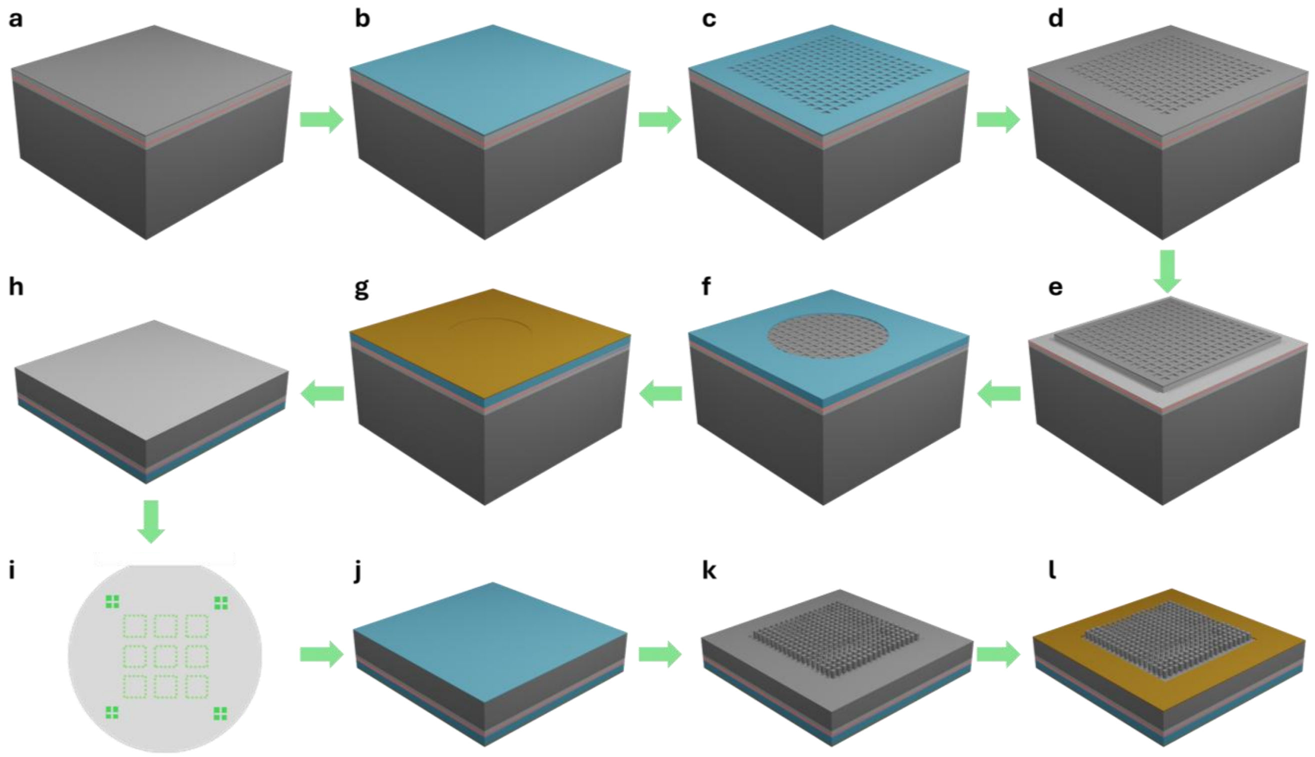

Figure S4. Fabrication process flow of the monolithically integrated metasurface-PCSEL device. a. Starting from an epitaxial (epi) wafer. **b.** Deposition of a Si_3N_4 hard mask via PECVD at 300 °C for photonic crystal (PhC) structure definition. **c.** Patterning of the PhC structure using electron-beam lithography, followed by dry etching of Si_3N_4 and GaAs. **d**. Removal of residual Si_3N_4 hard mask using buffered oxide etch (BOE). **e**. Mesa formation through UV lithography and GaAs dry etching. **f**. Deposition of a passivation layer and opening of the current confinement window using Si_3N_4 deposition, UV lithography, and wet etching. **g**. Deposition of Ti/Pt/Au for the p-contact metal via sputtering. **h**. Substrate thinning and surface polishing on the substrate side using chemical mechanical polishing (CMP), preparing for metasurface integration. **i**. Creation of alignment keys on the n-side using a double-side aligner, UV lithography, and GaAs dry etching to enable electron-beam lithography alignment. **j**. Deposition of a Si_3N_4 hard mask layer for metasurface patterning. **k**. Fabrication of the metasurface using electron-beam lithography and dry etching of Si_3N_4 and GaAs. **l**. Formation of the n-contact metal aperture through UV lithography, followed by Ni/Ge/Au deposition and annealing.

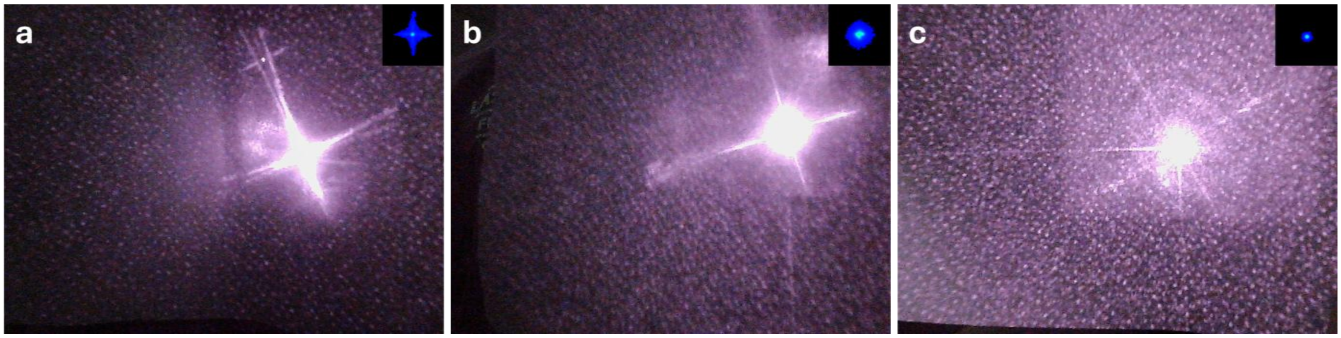

Figure S5. Structured-light projections generated using various PhC beam profiles illuminating the same metasurface hologram. a. Dot pattern projected by a cross-shaped PCSEL. Inset: far-field pattern of PCSEL Sample 1. **b.** Dot pattern projected by a standard PCSEL with a divergence angle of 2°. Inset: far-field pattern of PCSEL Sample 2. **c.** Dot pattern projected by a standard PCSEL with a divergence angle of 1°. Inset: far-field pattern of PCSEL Sample 3. Divergence angles for all PCSEL sources are summarized in Table S1.

Table S1. Divergence angles of different PCSEL samples.

	Sample 1	Sample 2	Sample 3
**FWHM (º)**	0.93	1.85	0.86
**1/e (º)**	1.11	2.2	1.03
**$1/e^2$ (º)**	2.85	3.1	1.42

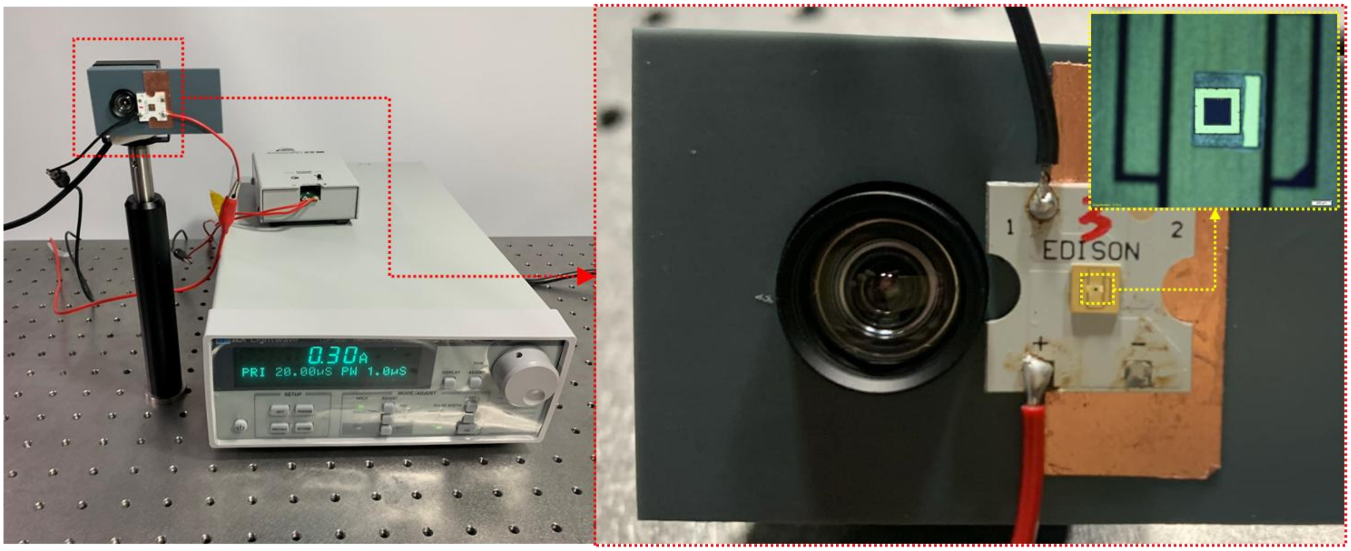

Figure S6. The real-world depth-sensing setup. It comprises a webcam (3200×720P, LRCP Luoke), the integrated device, a gray fixture, and a power supply (LDP-3830, Lightwave).

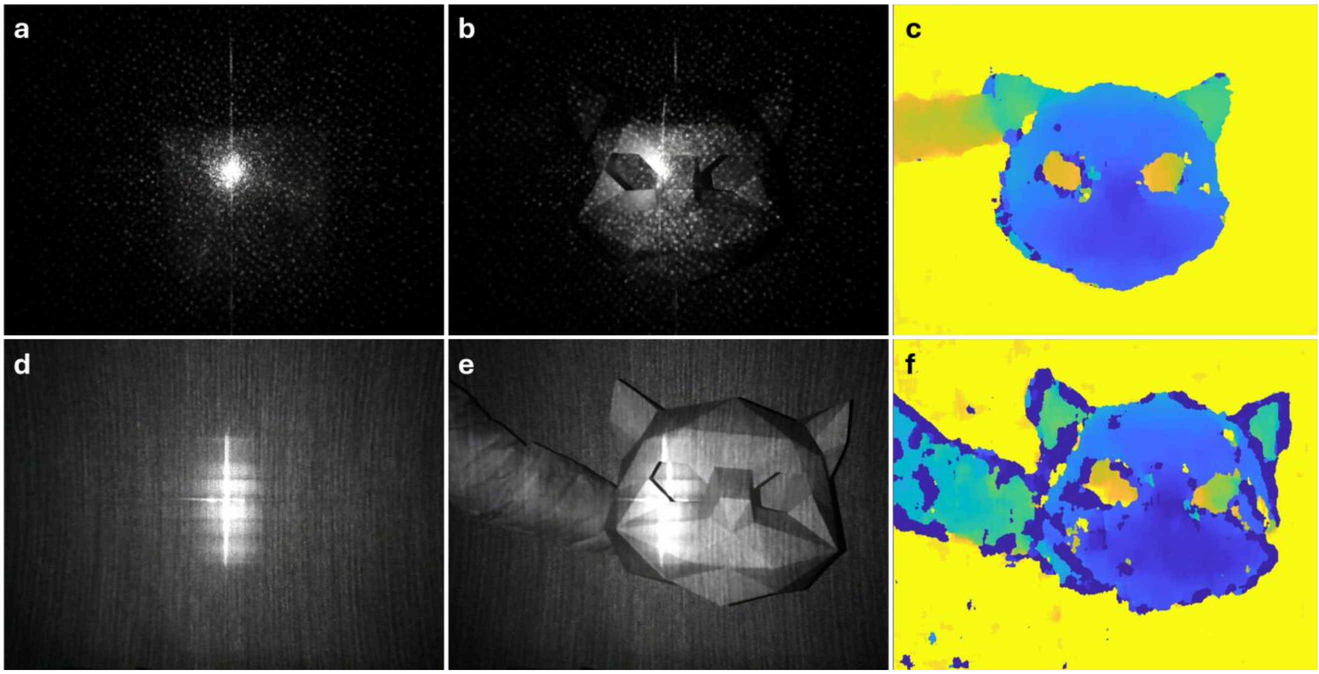

Figure S7. Comparison of structured-light projections between normal and abnormal cases. To minimize image saturation, the PCSEL operating current was set to 205 mA. The metasurface was designed to project a random dot projection. However, post-integration with the PCSEL, the emission exhibits an x-shaped (cross-shaped) distribution, indicating projection failure. **a.** Reference image of the normal structured-light projection generated by the non-monolithically integrated device (normal case). **b**. Object image under normal projection (target: cat helmet, ~40 cm from the camera). **c**. Reconstructed depth map from (a) and (b). **d**. Reference image of the abnormal structured-light projection generated by the monolithically integrated metasurface-PCSEL device (abnormal case). **e.** Object image under abnormal projection. **f.** Reconstructed depth map from (d) and (e).

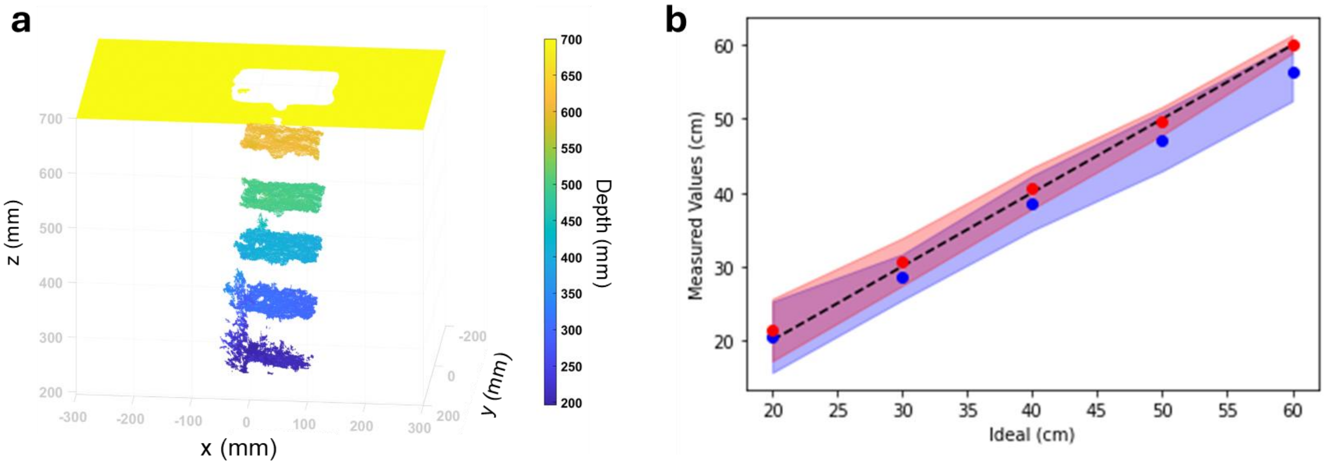

Figure S8. Comparison of depth-sensing performance between normal and abnormal structured-light projections. Structured-light projections used in this comparison are shown in Figures S7a (normal case) and S7d (abnormal case). **a.** Reconstructed point clouds of a planar board placed at varying distances from the non-monolithically integrated device (normal case). **b.** Depth error bands for the two cases. Red curves represent results from the non-monolithically integrated device (normal case), while blue curves represent results from the monolithically integrated metasurface-PCSEL device (abnormal case). Colored dots indicate the mean values, and the shaded areas represent one standard deviation, spanning ±0.5 standard deviation around the mean.
